# Feasibility, reproducibility and diagnostic usefulness of right ventricular strain by 2-dimensional speckle-tracking echocardiography in ARDS patients: the ARD strain study

**DOI:** 10.1186/s13613-020-0636-2

**Published:** 2020-02-13

**Authors:** Jérémie Lemarié, Charles-Henri Maigrat, Antoine Kimmoun, Nathalie Dumont, Pierre-Edouard Bollaert, Christine Selton-Suty, Sébastien Gibot, Olivier Huttin

**Affiliations:** 1 Service de Réanimation Médicale, Hôpital Central, CHRU de Nancy, 29 rue du Maréchal de Lattre de Tassigny, 54000 Nancy, France; 2 Service de Cardiologie, Institut Lorrain du Cœur et des Vaisseaux, CHRU de Nancy, 54511 Vandoeuvre-lès-Nancy, France; 3 Service de Médecine Intensive et Réanimation, Institut Lorrain du Cœur et des Vaisseaux, CHRU de Nancy, 54511 Vandoeuvre-lès-Nancy, France; 4 Plateforme d’Aide à la Recherche Clinique, Bâtiment Recherche, CHRU de Nancy, 54511 Vandoeuvre-lès-Nancy, France

**Keywords:** ARDS, Right ventricle, Speckle-tracking echocardiography, Feasibility, Reproducibility

## Abstract

**Background:**

Right ventricular (RV) function evaluation by echocardiography is key in the management of ICU patients with acute respiratory distress syndrome (ARDS), however, it remains challenging. Quantification of RV deformation by speckle-tracking echocardiography (STE) is a recently available and reproducible technique that provides an integrated analysis of the RV. However, data are scarce regarding its use in critically ill patients. The aim of this study was to assess its feasibility and clinical usefulness in moderate–severe ARDS patients.

**Results:**

Forty-eight ARDS patients under invasive mechanical ventilation (MV) were consecutively enrolled in a prospective observational study. A full transthoracic echocardiography was performed within 36 h of MV initiation. STE-derived and conventional parameters were recorded. Strain imaging of the RV lateral, inferior and septal walls was highly feasible (47/48 (98%) patients). Interobserver reproducibility of RV strain values displayed good reliability (intraclass correlation coefficients (ICC) > 0.75 for all STE-derived parameters) in ARDS patients. ROC curve analysis showed that lateral, inferior, global (average of the 3 RV walls) longitudinal systolic strain (LSS) and global strain rate demonstrated significant diagnostic values when compared to several conventional indices (TAPSE, S′, RV FAC). A RV global LSS value > − 13.7% differentiated patients with a TAPSE < vs > 12 mm with a sensitivity of 88% and a specificity of 83%. Regarding clinical outcomes, mortality and cumulative incidence of weaning from MV at day 28 were not different in patients with normal versus abnormal STE-derived parameters.

**Conclusions:**

Global STE assessment of the RV was highly achievable and reproducible in moderate–severe ARDS patients under MV and additionally correlated with several conventional parameters of RV function. In our cohort, STE-derived parameters did not provide any incremental value in terms of survival or weaning from MV prediction. Further investigations are needed to evaluate their theranostic usefulness.

*Trial registration* NCT02638844: NCT

## Background

Right ventricular (RV) dysfunction in acute respiratory distress syndrome (ARDS) is common and several mechanisms, including elevated pulmonary vascular tone, sepsis-induced cardiac dysfunction and positive-pressure ventilation, can combine to induce RV failure [1]. Thus, assessment of RV function is part of ARDS patient care [2]. Cardiac MRI allows highly accurate and reliable measurement of RV ejection fraction and is currently the gold standard imaging technique for RV assessment [3]. However, echocardiography is more practical in the unstable patient because it can be performed quickly at the bedside in a safer environment. There is currently significant interest for echocardiographic strain measurement derived from 2D speckle tracking imaging (STE), as it provides an objective quantification of myocardial mechanical function with unrivalled sensitivity. Hence, RV strain is now recommended as part of any comprehensive multiparametric assessment of RV function [4, 5]. However, data are scarce regarding its usefulness in mechanically ventilated patients in general and in ARDS patients in particular.

The main aims of the present investigation were to assess the feasibility and reproducibility of RV 2D STE-derived parameters in ARDS patients under invasive ventilation and to compare their diagnostic and prognostic role with conventional parameters of RV function assessment.

## Methods

### Patients

We performed a prospective observational study involving patients admitted to two medical intensive care units (ICU) at University Hospital of Nancy, France (Unit 1 from January 2016 to July 2017; Unit 2 from November 2016 to July 2017). Consecutive patients were eligible if they fulfilled the following criteria: [1] adult patients who met the Berlin definition criteria for moderate to severe ARDS, [2] were predicted to be under invasive mechanical ventilation (MV) for > 48 h, and [3] were expected to undergo a complete echocardiography from 1 to 36 h after MV initiation. Exclusion criteria were (i) vulnerable patients under curatorship or guardianship; (ii) a history of chronic respiratory failure (long-term oxygen therapy or non-invasive ventilation) or (iii) chronic right ventricular failure or (iv) chronic heart failure with LVEF < 35% or (v) severe valvular heart disease and (vi) extracorporeal membrane oxygenation started before echocardiography assessment. The study was approved by the institutional ethics committee of the French Intensive Care Society (decision CE SRLF15-39) which waived the need for patient’s consent. Written and oral information about the study was given to the patients and/or their next of kin. The trial was registered on clinicaltrials.gov under reference NCT02638844. We also included a cohort of control patients who were mechanically ventilated for airway protection only, without hypoxemia.

### Data collection

Baseline demographic characteristics, clinical characteristics, ventilator settings, and arterial blood gases at the time of echocardiography, SAPS II and SOFA severity scores, outcome (need for supportive therapies, ventilator-free days at day 28 (D28), mortality at D28 and D90) were collected.

### Echocardiography

Complete transthoracic echocardiography was performed with patients in the left lateral decubitus position whenever safely achievable or in supine position, using a Vivid S6 echocardiograph (GE Medical Systems, Milwaukee, WI) with simultaneous ECG recording, as previously described [6]. M-mode and 2D images were acquired by two physicians (JL and CHM, cardiologists with expert competency in advance critical care echocardiography and 2D-STE) in cine-loop format from three consecutive beats and analysed offline (EchoPAC version 201, GE Vingmed Ultrasound AS) in accordance with existing guidelines, after digital transfer on the institutional storage server [4].

### Right ventricular function

#### Conventional parameters

Right ventricle fractional area change (RVFAC), tricuspid annular plane systolic excursion (TAPSE), Doppler tissue imaging (DTI)-derived tricuspid lateral annular systolic velocity (S′), right ventricle/left ventricle (RV/LV) ratio were measured from a long-axis 4-chamber view. We also used a parameter that combined a RV/LV ratio > 0.6 and at least another abnormal conventional parameter among TAPSE < 12 mm and/or S′ < 11.5 cm s^−1^ and/or RVFAC < 35%, based on a previous publication in ventilated ARDS patients [7]. The same analyses were performed using classic cut-off values for non-ventilated patients (TAPSE < 17 mm and S′ < 9. 5 cm s^−1^).

#### 2D-STE

Cine loops from RV-focused 4- and 2-chamber apical views were recorded. The frame rate ranged between 50 and 80 frames/s. We recorded ≥ 3 consecutive cardiac cycles, encompassing approximately one respiratory cycle. Three RV walls were analysed: lateral and septal walls from 4-chamber view and inferior wall from 2-chamber view (obtained by rotating the probe counter-clockwise with an angle of 90° from the 4-chamber view focused on the RV). Each wall was divided into three segments (basal, mid-ventricular, and apical). Longitudinal systolic strain (LSS) is calculated as the percentage of systolic shortening of a myocardial wall from base to apex, while longitudinal strain rate (LSR) is the rate of this shortening. Peak LSS and LSR were measured using the Q-Analysis add-on included in EchoPAC software. The endocardial border of the RV wall was manually traced using a point-to-click approach. An automatic epicardial border tracing was then generated by the software, thus creating a region of interest that could be manually adjusted to the myocardial wall thickness to encompass the endocardium and the epicardium (usually 5 mm). The pericardium was excluded from the region of interest. For each segment, LSS and LSR were calculated as the average of both endocardial and epicardial layers. Tracking quality was automatically scored as either acceptable or unacceptable and further manually validated based on traces appearance. Segments without adequate tracking were excluded from further analysis. Global LSS and LSR were calculated as the average of septal, lateral and inferior LSS and LSR values, respectively. For each patient, STE-derived parameters were repeated and averaged on three consecutive cycles by two operators unaware of the measurements of the other operator.

### Statistical analysis

Continuous data are presented as mean ± standard deviation (SD) for normally distributed variables and as median [25,75 percentiles] for non-normally distributed variables. Categorical data are presented as frequencies and percentages. Between-group differences were assessed using the unpaired Student’s *t* test or Mann–Whitney U test for normally and non-normally distributed continuous variables, respectively, and by Chi-square test for categorical data. The association of STE-derived variables with conventional RV parameters was assessed by use of a Pearson correlation analysis. As there is no gold-standard parameter for RV assessment by echocardiography, we used receiver operating characteristic (ROC) curves to evaluate the diagnostic performance of STE-derived parameters in discriminating abnormal RV function among several conventional parameters using previously published threshold values. Interobserver reproducibility of STE-derived parameters was assessed in all patients. Intraclass correlation (ICC) coefficients were used to explore reproducibility of echocardiography measurements (single measurement, absolute agreement, 2-way mixed-effects model). Kaplan–Meier curves were plotted to assess survival and time to weaning from MV at day 28 depending on the presence or absence of RV dysfunction and were compared by means of the log-rank test. To show a sensitivity and a specificity of 0.90 (marginal error 0.1), considering a prevalence of right ventricular dysfunction of 30%, a minimum sample size of 49 patients was required to provide the study with 80% power and 95% confidence level. All statistical analyses were performed with SPSS version 22 or Graphpad Prism version 7.04. All statistical tests were two sided, and a *p* value < 0.05 was considered to indicate statistical significance.

## Results

### Population

**Fig. 1:**
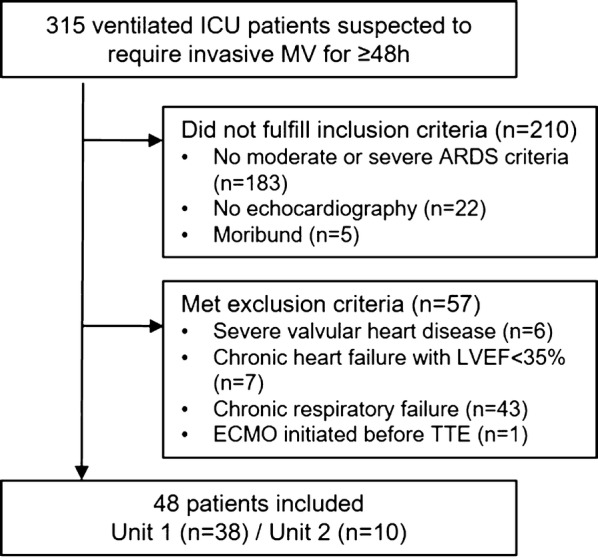
Flowchart of the cohort of ARDS patients. *ICU* intensive care unit, *MV* mechanical ventilation, *ARDS* acute respiratory distress syndrome, *LVEF* left ventricular ejection fraction; *ECMO* extracorporeal membrane oxygenation

During the study period, 315 ventilated patients were screened, among whom 48 patients (male 27 (56%), mean age 61 ± 17 years old) were included in the ARDS cohort (Fig. 1). ARDS patients’ characteristics are presented in Table 1. The main cause for ARDS was pneumonia (*n* = 32 (67%): *Streptococcus pneumoniae*, *n* = 6; influenza, *n* = 3; no documentation, *n* = 16; aspiration pneumonia, *n* = 8). Of note, no patient had previously undergone open-chest cardiac surgery.

**Table 1:** ARDS patients’ characteristics

Parameters	ARDS cohort (*n* = 48)	D28 ARDS survivors (*n* = 36)	D28 ARDS non-survivors (*n* = 12)	Comparison survivors vs non-survivors
Sex (M, %)	27 (56%)	22 (61.1%)	5 (41.7%)	0.24 (Chi^2^)
Age (years)	61 ± 17	58 ± 17	68 ± 14	0.25
SOFA	8 [7;11]	8 [7;11]	10 [8;14]	0.09 (MW)
SAPS II	57 ± 15	56 ± 16	60 ± 14	0.49
Weight (kg)	75.7 ± 20.3	79.5 ± 21.2	64.4 ± 12.1	0.02
Circulatory parameters				
SAP/MAP/DAP mmHg	114 ± 19/77 ± 10/57 ± 9	114 ± 16/78 ± 10/59 ± 9	114 ± 28/72 ± 11/51 ± 8	0.99/0.10/0.01
Heart rate bpm	95 ± 22	95 ± 23	95 ± 19	0.97
Sinus rhythm	42 (87.5%)	31 (86.1%)	11 (91.7%)	0.61 (Chi^2^)
Ventilator settings				
Tidal volume, ml/kg of IBW	6.9 ± 1.1	6.9 ± 1.2	6.8 ± 0.8	0.74
PEEP cmH_2_O	10.6 ± 2.9	9.8 ± 2.6	10.2 ± 2.9	0.65
Autopeep cmH_2_O	0.8 ± 1.2	0.9 ± 1.3	0.5 ± 0.7	0.31
Plateau pressure cmH_2_O	23.2 ± 4.4	22.6 ± 4.3	24.3 ± 4.5	0.25
Driving pressurecmH_2_O	12.4 ± 4.1	11.8 ± 4.1	13.7 ± 3.9	0.19
Respiratory rate/min	24.0 ± 3.5	23.3 ± 3.4	25.5 ± 3.8	0.01
Arterial blood gases				
FiO_2_ (%)	70 ± 21	67 ± 19	79 ± 25	0.10
pH	7.31 ± 0.14	7.34 ± 0.13	7.23 ± 0.16	0.01
pCO_2_ mmHg	45.6 ± 11.1	45.6 ± 12.1	45.7 ± 7.4	0.96
pO_2_ mmHg	83.6 ± 32.8	81.1 ± 24.1	91.3 ± 51.7	0.36
HCO_3_-mmol	22.1 ± 6.1	23.1 ± 5.9	19.1 ± 5.8	0.05
SaO_2_%	93.5 ± 5.0	94.0 ± 2.8	91.9 ± 8.9	0.20
Lactate mmol	2.1 ± 2.4	1.4 ± 0.9	3.9 ± 4.0	0.002
PaO_2_:FiO_2_ ratio	126 ± 48	127 ± 46	123 ± 55	0.77
Treatments in ICU				
Vasopressor support	32 (66.7%)	21 (58.3%)	11 (91.7%)	0.03 (Chi^2^)
Inotropic support	6 (12.5%)	2 (5.6%)	4 (33.3%)	0.01 (Chi^2^)
Neuromuscular blockade > 24 h	30 (62.5%)	25 (69.4%)	9 (75%)	0.71 (Chi^2^)
Prone positioning	18 (37.5%)	15 (41.7%)	3 (25%)	0.30 (Chi^2^)
Inhaled NO	6 (12.5%)	3 (8.3%)	3 (25%)	0.13 (Chi^2^)
VV ECMO	2 (4.2%)	1 (2.8%)	1 (8.3%)	0.10 (Chi^2^)
Renal replacement therapy	12 (25%)	9 (25%)	3 (25%)	1.00 (Chi^2^)
Outcome				
Mortality D28 n (%)	12 (25%)	–	–	
Mortality D90 n (%)	14 (29.2%)	–	–	
D28 ventilator-free days	20.0 [1.3; 23.0]	20.5 [14.3; 23.8]	0 [0; 5.25]	0.003 (MW)

Median [interquartile range]; mean ± standard deviation; number (percentage)

Statistical test: Student’s *t* test unless specified: MW, Mann–Whitney or Pearson Chi^2^

*SOFA* sequential organ failure assessment, *SAPS II* Simplified Acute Physiology Score II, *SAP–MAP–DAP* systolic–mean–diastolic arterial pressure, *IBW* ideal body weight; PEEP: positive end-expiratory pressure, *NO* nitric oxide, *VV ECMO* veno-venous extracorporeal membrane oxygenation

Control patients’ characteristics and comparison with ARDS patients are presented in Additional file 1: Table S1. Respiratory support for airway protection was related to neurological failure in all control patients. When compared to control patients, ARDS patients had higher airway pressure (PEEP 10.6 cmH_2_O ± 2.9 vs 4.7 ± 0.5 and plateau pressure 23.2 cmH_2_O ± 4.4 vs 14.8 ± 0.5, *p *≤ 0.001 for both) and lower PaO_2_:FiO_2_ ratio (126 ± 48 vs 323 ± 92, *p *< 0.001).

Among ARDS patients, 12 (25%) died before day 28 and 14 (29.2%) before day 90. Differences between D28 survivors and non-survivors are shown in Table 1. D28 non-survivors required more vasopressor and inotropic support, had lower arterial pH, higher arterial lactate and respiratory rate. Severity scores at presentation (SOFA and APACHE II) were not significantly different.

### Feasibility and reproducibility

A full STE examination including recordings of 3 cardiac cycles for each of the 3 RV walls by at least one operator was achieved in 98% of ARDS patients and 100% of control patients (Additional file 1: Table S2). All the conventional RV parameters were also successfully obtained for all of both ARDS and control patients.

Interobserver reproducibility was assessed for STE-derived parameters as well as for conventional indices (Additional file 1: Table S3). In ARDS patients, RV global LSS and global LSR demonstrated the highest ICC coefficients among all STE-derived parameters [0.87 (0.72; 0.93) and 0.91 (0.84; 0.95), *p* < 0.001 for both, respectively] while RV FAC showed weak reproducibility between the 2 observers [ICC 0.57 (0.35; 0.73), *p* = 0.001]. ICC for TAPSE and S′ were 0.88 (0.78; 0.93) and 0.94 (0.89; 0.97), respectively.

### Echocardiography parameters

Main echocardiography parameters are listed in Table 2 (ARDS cohort and comparison between D28 survivors and non-survivors) and in Additional file 1: Table S1 (control patients and their comparison with ARDS patients). When compared to controls, LSS from all three RV walls was significantly impaired in ARDS patients. Conventional RV parameters were also significantly decreased in ARDS patients. Within the ARDS cohort, none of the right or left ventricular parameters differed between D28 survivors and non-survivors, except TAPSE which was significantly more impaired in non-survivors (19.6 mm ± 4.7 vs 15.8 ± 5.0, *p* = 0.02). Of note, there was also a trend towards a lower LVEF in non-survivors (56.8% ± 11.5 vs 50.0 ± 10.7, *p* = 0.09).

**Table 2:** Echocardiography parameters in ARDS patients

Parameters	ARDS cohort (*n* = 48)	D28 ARDS survivors (*n* = 36)	D28 ARDS Non-survivors (*n* = 12)	Comparison survivors vs non-survivors
RV STE-derived parameters				
RV inferior LSS%	− 19.2 ± 7.0	− 19.1 ± 6.6	− 19.8 ± 8.2	0.76
RV lateral LSS%	− 19.9 ± 6.4	− 20.3 ± 6.1	− 18.7 ± 7.5	0.49
RV septal LSS%	− 13.9 ± 4.1	− 13.9 ± 3.6	− 13.6 ± 5.3	0.80
RV global LSS%	− 17.7 ± 4.9	− 17.7 ± 4.5	− 17.4 ± 6.0	0.82
RV global LSR s^−1^	− 1.37 ± 0.46	− 1.36 ± 0.45	− 1.39 ± 0.52	0.87
RV conventional parameters				
TAPSE mm	18.7 ± 5.0	19.6 ± 4.7	15.8 ± 5.0	*0.02*
RV FAC%	39.5 ± 9.2	39.5 ± 9.0	39.4 ± 9.6	0.98
S’ cm s^−1^	12.7 ± 5.4	13.2 ± 5.9	11.1 ± 3.3	0.13
LV parameters				
LVEF (Simpson’s method)%	55.2 ± 11.6	56.8 ± 11.5	50.0 ± 10.7	0.09
Cardiac output l min^−1^	5.7 ± 1.8	5.8 ± 1.5	5.4 ± 2.7	0.61
E/e’	8.2 ± 2.9	8.2 ± 2.7	8.1 ± 3.5	0.97

Mean ± standard deviation

Italic font indicates significant difference (*p* < 0.05)

*RV/LV* right/left ventricle, *STE* speckle-tracking echocardiography, *LSS* longitudinal systolic strain, *LSR* longitudinal systolic strain rate, *TAPSE* tricuspid annular plane systolic excursion, *FAC* fractional area change, *S*′ peak systolic velocity of tricuspid annulus by pulsed wave Doppler tissue imaging, *EF* ejection fraction, *E/e*′ ratio between early mitral inflow velocity and mitral annular early diastolic velocity

### Comparison between STE-derived and conventional parameters of RV function

**Fig. 2:**
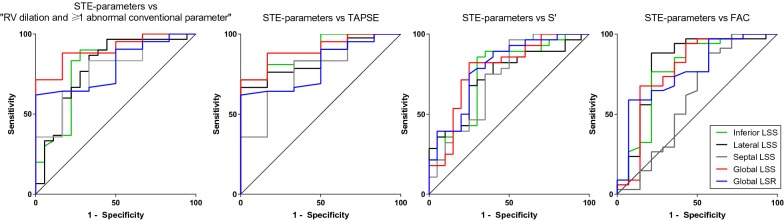
Diagnostic value of STE-derived parameters in discriminating RV dysfunction diagnosed by impaired conventional parameters. *STE* speckle-tracking echocardiography, *RV* right ventricle, *LSS* longitudinal systolic strain, *LSR* longitudinal systolic strain rate, *TAPSE* tricuspid annular plane systolic excursion, *FAC* fractional area change, *S*′ peak systolic velocity of tricuspid annulus by pulsed wave Doppler tissue imaging

To evaluate the ability of RV strain parameters to prognosticate RV dysfunction during ARDS, we compared them to currently in-use conventional parameters. Firstly, we used previously published cut-off thresholds in ARDS ventilated patients (i.e. RV FAC < 35%, TAPSE < 12 mm and S′ < 11.5 cm s^−1^) for ROC curve analysis [7] (Table 3 and Fig. 2). RV global LSS provided the highest diagnostic performance to discriminate between patients with TAPSE < 12 mm versus > 12 mm (area under the ROC curve (AUROC) 0.905; 95% CI [0.805–1.00]; *p* = 0.002). A threshold of − 13.7% differentiated patients with TAPSE < 12 mm versus > 12 mm with a sensitivity of 88% and a specificity of 83%. Whichever RV wall considered, strain parameters performed better in classifying patients with TAPSE < 12 mm than any other abnormal conventional parameter (S′, RVFAC or RV dilation plus at least another abnormal conventional parameter. Secondly, we repeated these analyses while using widely used cut-off thresholds derived from large cohorts of non-ventilated patients (EACVI/ASE guidelines, i.e. TAPSE < 17 mm and S′ < 9.5) [4]. Once again, RV global LSS provided the highest diagnostic performance to discriminate between patients with normal vs abnormal TAPSE (i.e. > vs < 17 mm) (AUROC 0.759; 95% CI [0.614–0.904]; *p* = 0.002) (Additional file 1: Table S4).

**Table 3:** Diagnostic value of STE-derived parameters in discriminating RV dysfunction diagnosed by conventional parameters

STE-derived parameters of RV function	TAPSE (cut-off 12 mm)			S’ (cut-off 11.5 cm/s)			RV FAC (cut-off 35%)			ED RV: LV > 0.6 and at least 1 abnormal conventional parameter		
	AUROC	95% CI	*p*	AUROC	95% CI	*p*	AUROC	95% CI	*p*	AUROC	95% CI	*p*
RV inferior LSS	0.893	0.783–1.00	0.002	0.75	0.603–0.897	0.003	0.776	0.609–0.944	0.003	0.814	0.675–0.953	0.001
RV lateral LSS	0.849	0.724–0.974	0.006	0.736	0.593–0.879	0.006	0.811	0.648–0.974	0.001	0.793	0.650–0.935	0.001
RV septal LSS	0.778	0.583–0.973	0.029	0.727	0.575–0.879	0.008	*0.604*	*0.400–0.809*	*0.262*	*0.637*	*0.469–0.805*	*0.115*
RV global LSS	0.905	0.805–1.00	0.002	0.779	0.639–0.919	0.001	0.777	0.605–0.949	0.003	0.806	0.668–0.943	0.001
RV GLOBAL LSR	0.802	0.650–0.953	0.018	0.789	0.656–0.922	0.001	0.769	0.618–0.920	0.004	0.812	0.693–0.931	0.001

Underline and italic indicates significant difference (*p* < 0.05)

*AUROC* area under receiver operating characteristic, *RV* right ventricle, *STE* speckle-tracking echocardiography, *LSS* longitudinal systolic strain, *LSR* longitudinal systolic strain rate, *TAPSE* tricuspid annular plane systolic excursion, *FAC* fractional area change, *S′* peak systolic velocity of tricuspid annulus by pulsed wave Doppler tissue imaging, *ED RV:LV* end diastolic right ventricular over left ventricular diameter ratio

Correlations between STE-derived and conventional parameters of RV function in ARDS patients are shown in Additional file 1: Table S5.

### Clinical diagnostic value of STE-derived parameters

#### Correlation with clinical parameters

We next evaluated correlations between RV echocardiography parameters and relevant hemodynamic and respiratory variables (Table 4). All LSS parameters correlated with the duration of inotropic support, as well as TAPSE. Among STE-derived parameters, all but RV inferior LSS significantly correlated with LVEF and cardiac output, while TAPSE and S′, but not RV FAC, also showed significant correlations with these two parameters. Regarding respiratory variables, no correlation was found between PEEP or driving pressure levels and neither of the RV parameters. However, there was a significant correlation between PaO_2_:FiO_2_ ratio and RV Inferior, Lateral and Global LSS.

**Table 4:** Correlation coefficients (Pearson) between echocardiography and clinical variables

Echocardiography parameters	Hemodynamic				Respiratory		
	Duration of vasopressor support	Duration of inotropic support	LVEF	Cardiac output	PaO2: FiO2 ratio	PEEP	Driving pressure
RV STE-derived parameters							
RV inferior LSS %	*− 0.119*	0.363*	*− 0.155*	*− 0.259*	*− *0.403*	*0.082*	*− 0.066*
RV lateral LSS%	*− 0.160*	0.384*	*− *0.301*	*− *0.379*	*− *0.304*	*− 0.042*	*− 0.187*
RV septal LSS%	*0.133*	0.465*	*− *0.496*	*− *0.395*	*− 0.098*	*0.044*	*− 0.296*
RV global LSS%	*− 0.090*	0.471*	*− *0.335*	*− *0.390*	*− *0.353*	*0.033*	*− 0.194*
RV global LSR s^−1^	*− 0.245*	*0.320*	*− *0.294*	*− *0.457*	*− 0.175*	*− 0.144*	*− 0.037*
RV conventional parameters							
TAPSE mm	*− 0.143*	*− *0.489*	0.288*	0.300*	*0.098*	*− 0.138*	*0.034*
RV FAC%	*0.009*	*− 0.251*	*0.014*	*0.199*	*0.026*	*0.154*	*− 0.145*
S’ cm s^−1^	*− 0.036*	*− 0.288*	0.357*	0.415*	*0.162*	*− 0.024*	*0.004*

Underline and * indicate significant correlation (*p* < 0.05); italic font: no significant correlation

*RV/LV* right/left ventricle, *STE* speckle-tracking echocardiography, *LSS* longitudinal systolic strain, *LSR* longitudinal systolic strain rate, *TAPSE* tricuspid annular plane systolic excursion, *FAC* fractional area change, *S′* peak systolic velocity of tricuspid annulus by pulsed wave Doppler tissue imaging, *EF* ejection fraction, *PEEP* positive end-expiratory pressure

We finally assessed the probability of survival as well as the probability of being weaned from MV at D28 depending on the presence of an abnormal echocardiography parameter. Cut-off values were based on previously published thresholds for conventional parameters and/or derived from control patients’ characteristics for STE-derived parameters (mean ± 2SD) (Additional file 1: Table S6). The cumulative probability of being weaned from MV was not different between ARDS patients presenting with or without RV dysfunction, whichever parameter considered (Table 5). The same held true for survival, except for ARDS patients with severely decreased TAPSE < 12 mm (*n* = 6) who showed a significantly worse probability of survival (18.3 days ± 3.4 vs 25.5 ± 0.9, log-rank test *p* = 0.005). However, using a more widely accepted cut-off value (abnormal TAPSE < 17 mm, *n* = 18), the difference in survival was no longer found (23.4 days ± 1.8 vs 25.3 ± 1.8, log-rank test *p* = 0.31).

**Table 5:** Log-rank test of survival and cumulative incidence of weaning from MV depending on the presence or absence of an abnormal RV echocardiography parameter

Echocardiography parameters	Mean survival time, days			Mean time to weaning from MV, days		
	Patients with a normal parameter	Patients with an abnormal parameter	*p*	Patients with a normal parameter	Patients with an abnormal parameter	*p*
Abnormal cut-off values based on literature (Ref. [7])						
TAPSE < 12 mm	25.5 ± 0.9	18.3 ± 3.4	0.005*	13.6 ± 1.4	13.6 ± 2.4	*0.32*
RV FAC < 35%	25.5 ± 1.0	22.4 ± 2.2	*0.24*	13.4 ± 1.6	15.8 ± 2.5	*0.43*
S′ < 11.5 cm/s	25.1 ± 1.3	23.9 ± 1.6	*0.21*	13.7 ± 1.8	13.9 ± 1.8	*0.75*
Abnormal cut-off values based on 2015 EACVI/ASE guidelines (Ref. [4])						
TAPSE < 17 mm	25.3 ± 1.1	23.4 ± 1.8	*0.31*	13.6 ± 1.7	14.9 ± 2.3	*0.57*
RV FAC < 35%	25.5 ± 1.0	22.4 ± 2.2	*0.24*	13.4 ± 1.6	15.8 ± 2.5	*0.43*
S′ < 9.5 cm/s	24.7 ± 1.1	24.2 ± 1.8	*0.57*	14.5 ± 1.5	10.2 ± 2.0	*0.55*
Abnormal cut-off values derived from control patients (mean ± 2SD)						
RV STE-derived parameters						
RV inferior LSS > − 17.6%	25.3 ± 1.2	23.7 ± 1.6	*0.57*	13.7 ± 1.8	14.6 ± 2.0	*0.78*
RV lateral LSS > − 17.8%	25.1 ± 1.1	23.9 ± 1.7	*0.94*	15.8 ± 1.9	11.4 ± 1.5	*0.13*
RV septal LSS > − 11.1%	25.4 ± 1.1	22.6 ± 2.1	*0.24*	14.5 ± 1.6	11.2 ± 1.5	*0.68*
RV global LSS > − 17.4%	24.2 ± 1.5	25.0 ± 1.3	*0.44*	14.8 ± 2.2	13.5 ± 1.6	*0.60*
RV global LSR > − 1.02	25.0 ± 1.0	22.7 ± 2.8	*0.48*	14.2 ± 1.4	14.6 ± 3.8	*0.97*

Underline and * indicate significant correlation (*p *< 0.05); italic font: no significant correlation

*RV* right ventricle, *STE* speckle-tracking echocardiography, *LSS* longitudinal systolic strain, *LSR* longitudinal systolic strain rate, *TAPSE* tricuspid annular plane systolic excursion, *FAC* fractional area change, *S′* peak systolic velocity of tricuspid annulus by pulsed wave Doppler tissue imaging, *MV* mechanical ventilation

## Discussion

The main findings of the present study are that 2D speckle tracking imaging of the right ventricle is feasible in ARDS patients under invasive MV, is reproducible and is able to discriminate patients with RV dysfunction when compared to conventional parameters.

Right ventricular function evaluation by echocardiography is challenging. The RV has a unique crescent shape which precludes simple quantification of its size and function [4]. Moreover, in ICU, adequate acoustic window is often impaired by mechanical ventilation and suboptimal patient positioning [8]. These obstacles, as well as the predominance of longitudinal-oriented muscle fibres, have led to the widespread use of parameters that estimate RV function based only on its lateral wall longitudinal motion (such as TAPSE or S′) [9]. Indices that measure the global function (such as RV FAC) have been shown to have lower feasibility [7]. However, continuous evolution of echocardiographic technology has greatly improved image quality. In our study, we found that conventional and STE-derived parameters were highly feasible. For instance, RV lateral LSS, the most widely used strain parameter, was measured on at least one cardiac cycle in all our patients from both ARDS and control cohorts. Two previous studies assessing RV lateral LSS in critically ill ventilated patients have reported feasibility rate of 83% (20/24) and 93% (28/30) [10, 11]. Our high feasibility rate could be explained by the fact that we tried to perform TTE examination with patients in the left lateral decubitus position, which proved to be safe despite the potential modification of airway and hemodynamic parameters. Not only were strain-derived parameters highly feasible, but also reproducible. Interobserver reproducibility assessed by ICC was > 0.75 for all LSS indices, which indicates good reliability [12], much higher than for RV FAC. The same findings were recently reported in ARDS patients by Garcia-Montilla et al., with high ICC coefficients for RV lateral strain but a low interobserver agreement for RVFAC [13]. This could be explained by the software-based automated tracing of the endocardial border, which limits operator dependency.

To date, there are no gold standard echocardiographic indices for RV function assessment. Many indices are available, with their own strengths and limitations [9]. Furthermore, for each index, there is no definite cut-off value to define RV dysfunction, especially in ventilated ICU patients in whom load conditions and positive-pressure ventilation can substantially alter “normal” values. Thus, we decided to compare STE-derived parameters to several currently in-use RV conventional indices. Apart from septal LSS, we found significant correlations between strain parameters and TAPSE, S′ and RV FAC. Using ROC curves, we also found that these strain parameters allowed to significantly differentiate patients with normal from those with abnormal RV function (defined by previously published cut-offs values for TAPSE, S′, RV FAC and a composite index). Based on our control cohort, we also provided cut-off values for abnormal LSS of all three RV walls, as well as for global LSS and LSR parameters. We hope these preliminary data will lay the groundwork for further in-depth assessment of these promising tools in ICU patients.

Unlike the majority of studies in the field of RV strain, we not only analysed strain values in the lateral wall but also in the septal and inferior walls [14]. Left ventricular contraction is believed to account for 20 to 40% of RV systolic pressure and outflow [15]. This contribution increases in conditions of high RV afterload. Due to the oblique orientation of its muscular fibres, the contraction of the interventricular wall induces a twist of the RV cavity that produces ejection. When pulmonary vascular resistance increases, the RV must rely upon this intrinsic twisting function to maintain output [16]. As high RV afterload is likely to occur in ARDS patients, incorporating septal strain values could help best describe RV function in this subset of patients. RV inferior wall strain has been even less explored than septal strain. Yet, its feasibility was high in our cohorts and it has been shown to add incremental value to RV function assessment in situations of regional wall motion abnormalities [6].

Recently, a group of eleven international experts in the field of critical care ultrasonography raised some important unanswered questions related to CCUS which should be trialled [17]. One of these questions was the usefulness of speckle-tracking strain and strain rates in evaluating heart–lung interactions with focus on adverse ventricular interdependence effects of tidal volume and PEEP in ARDS. In the present investigation, we could not find any correlation between STE-derived parameters and PEEP or driving pressure, but pressure levels were moderate (PEEP 10.6 mmHg ± 2.9 and driving pressure 12.4 ± 4.1). However, we did find significant correlations between RV Global LSS and the duration of inotropic support, cardiac output or PaO_2_:FiO_2_ ratio, suggesting that speckle-tracking strain imaging could be useful in helping fine-tune ventilator settings on a short timescale to minimize heart–lung and ventricular interactions.

### Limitations

We did not find any association between STE-derived parameters and the probability of survival or of being weaned from MV at D28, while others have previously reported such an association in ARDS patients [10]. Some explanations could be a greater heterogeneity in our patients’ presentation or a lower mortality rate. Either way, it is unlikely that one single measurement at one time-point could accurately predict long-term outcomes in the complex setting of critical care. STE has the distinctive ability to detect subtle changes in systolic function. Thus, as discussed above, using these highly sensitive tools to serially assess RV function during a short therapeutic intervention could be more useful. For instance, RV lateral LSS has already been studied during recruitment manoeuvres in a large animal pre-clinical model and in ARDS patients [11, 18]. Both these studies found significant impairment in RV lateral LSS that were not detected by conventional parameters while increasing PEEP levels.

Based on our findings, one could argue that TAPSE, on top of being easy to perform, was also significantly different between survivors and non-survivors, thus combining time-efficiency and diagnostic accuracy. However, TAPSE has been shown to be correlated with left ventricular function, thus reflecting not only the presence of right, but also left ventricular failure [19]. Moreover, we predefined a very low abnormal cut-off value of < 12 mm. It induced a very low number of patients with abnormal TAPSE (*n* = 6/48), which is known to lead to the “rare event problem” where specificity disproportionately drives accuracy [20]. Indeed, when we assessed the probability of survival with or without abnormal TAPSE using a more relevant cut-off derived (i.e. 17 mm), we could not find any difference between the two groups.

## Conclusion

Global STE assessment of the RV was highly achievable and reproducible in moderate–severe ARDS patients under invasive MV and additionally correlated with several conventional parameters of RV function. In our cohort, STE-derived parameters did not provide any incremental value in terms of survival or weaning from MV prediction. Further investigations are needed to evaluate their theranostic usefulness.

## Supplementary information

**Additional file 1.** Details about control patients’ characteristics, feasibility and inter-observer reproducibility of RV parameters, diagnostic value of STE-derived parameters and their correlation with conventional RV indices as well as the cut-off values derived from the control patients.

## Data Availability

The datasets used and analysed during the current study are available from the corresponding author on reasonable request.

## Abbreviations

2D: 2-Dimension
ARDS: Acute respiratory distress syndrome
DTI: Doppler tissue imaging
FAC: Fractional area change
IBW: Ideal body weight
ICC: Intraclass correlation
ICU: Intensive care unit
LSS: Longitudinal systolic strain
LSR: Longitudinal strain rate
LV: Left ventricle
MV: Mechanical ventilation
NO: Nitric oxide
PEEP: Positive end-expiratory pressure
RV: Right ventricle
S′: Peak systolic velocity of tricuspid annulus by pulsed wave Doppler tissue imaging
SAPS II: Simplified acute physiology score II
SAP–MAP–DAP: Systolic–mean–diastolic arterial pressure
SD: Standard deviation
STE: Speckle-tracking echocardiography
SOFA: Sequential organ failure assessment
TAPSE: Tricuspid annular plane systolic excursion
VV ECMO: Veno-venous extracorporeal membrane oxygenation
