# Supplementary material for: Feasibility, reproducibility and diagnostic usefulness of right ventricular strain by 2-dimensional speckle-tracking echocardiography in ARDS patients: the ARD strain study
Source: Ann Intensive Care. 2020 Feb 13;10:24. doi: 10.1186/s13613-020-0636-2 (PMC7018922; doi:10.1186/s13613-020-0636-2)
Supplement: Supplementary file 1 — Additional file 1. Details about control patients’ characteristics, feasibility and inter-observer reproducibility of RV parameters, diagnostic value of STE-derived parameters and their correlation with conventional RV indices as well as the cut-off values derived from the control patients. [file 13613_2020_636_MOESM1_ESM.pdf]

Table S1: Control patients'characteristics

Parameter	Control cohort (n=6)	ARDS cohort (n=48)	Comparison control vs ARDS patients
Sex (M,%)	4 (67%)	27 (56%)	0.24 (Chi^2)
Age (years)	51 ± 8	61 ± 17	0.16
SOFA	NA	8 [7;11]	--
APACHE II	NA	57 ± 15	--
Weight (kg)	NA	75.7 ± 20.3	--
Circulatory parameters			
• SAP/MAP/DAP mmHg	144 ± 16 / 96 ± 7 / 74 ± 7	114 ± 19 / 77 ± 10 / 57 ± 9	0.001 / <0.001 / <0.001
• Heart rate bpm	81 ± 19	95 ± 22	0.12
• Sinus rhythm	6 (100%)	42 (87.5%)	
Ventilator settings			
• Tidal volume, ml/kg of IBW	8.5 ± 3.16	6.9 ± 1.1	0.01
• PEEP cmH2O	4.7 ± 0.5	10.6 ± 2.9	<0.001
• autoPEEP cmH2O	0.1 ± 0.1	0.8 ± 1.2	0.19
• Plateau pressure cmH2O	14.8 ± 0.5	23.2 ± 4.4	0.001
• Driving pressure cmH2O	10.1 ± 3.0	12.4 ± 4.1	0.31
• Respiratory rate /min	18.5 ± 2.8	24.0 ± 3.5	<0.001
Arterial blood gases			
• FiO2 (%)	29 ± 6	70 ± 21	<0.001
• pH	7.47 ± 0.06	7.31 ± 0.14	0.01
• pCO2 mmHg	34.9 ± 3.7	45.6 ± 11.1	0.02
• pO2 mmHg	88.8 ± 19.0	83.6 ± 32.8	0.71
• HCO3- mmol	25.0 ± 3.8	22.1 ± 6.1	0.27
• SaO2 %	96.7 ± 2.4	93.5 ± 5.0	0.14
• Lactate mmol	1.0 ± 0.4	2.1 ± 2.4	0.34
• PaO2:FiO2 ratio	323 ± 92	126 ± 48	<0.001
Echocardiography			
• RV STE-derived parameters			
○ RV Inferior LSS %	-25.7 ± 4.1	-19.2 ± 7.0	0.03
○ RV Lateral LSS %	-27.7 ± 4.9	-19.9 ± 6.4	0.006
○ RV Septal LSS %	-19.5 ± 4.2	-13.9 ± 4.1	0.003
○ RV Global LSS %	-24.3 ± 3.4	-17.7 ± 4.9	0.002
○ Global systolic strain rate s^{-1}	-1.76 ± 0.37	-1.37 ± 0.46	0.06
• RV conventional parameters			
○ TAPSE mm	24.2 ± 3.3	18.7 ± 5.0	0.01
○ RV FAC %	47.0 ± 5.6	39.5 ± 9.1	0.05
○ S' cm/s	18.0 ± 2.2	12.7 ± 5.4	0.03
○ Acute cor pulmonale n (%)	0 (0%)	6 (12.5%)	0.36 (Chi^2)
• LV parameters			
○ LVEF (Simpson's method) %	64.1 ± 7.2	55.2 ± 11.6	0.07
○ Cardiac output l/min	9.2 ± 6.2	5.7 ± 1.8	0.003
○ E/e'	6.2 ± 1.7	8.2 ± 2.9	0.15

Median [interquartile range] ; mean ± standard deviation ; number (percentage)
Statistical test: Student t-test unless specified: Pearson Chi^2
SOFA = sequential organ failure assessment score; APACHE II = Acute Physiology And Chronic Health Evaluation II; SAP-MAP-DAP = systolic-mean-diastolic arterial pressure; IBW = ideal body weight; PEEP = positive end-expiratory pressure; RV/LV = right/left ventricle; STE = speckle tracking echocardiography; LSS = longitudinal systolic strain; TAPSE = Tricuspid annular plane systolic excursion; FAC = Fractional area change; S' = Peak systolic velocity of tricuspid annulus by pulsed wave Doppler tissue imaging; EF = ejection fraction; E/e' = ratio between early mitral inflow velocity and mitral annular early diastolic velocity.

Table S2: Feasibility

	ARDS patients	Control patients
Full STE examination (3 cardiac walls recorded with 3 cardiac cycles)		
• Operator 1 (JL)	81 (39)	83% (5)
• Operator 2 (CHM)	94% (45)	100% (6)
• At least one operator with full examination	98% (47)	100% (6)
3-wall STE examination (3 cardiac walls recorded with ≥ 1 cardiac cycle)		
• Operator 1	98% (47)	100% (6)
• Operator 2	100% (48)	100% (6)
RV inferior wall		
• 3 cardiac cycles		
○ Operator 1	92% (44)	83% (5)
○ Operator 2	100% (48)	100% (6)
• ≥ 1 cardiac cycle		
○ Operator 1	98% (47)	100% (6)
○ Operator 2	100% (48)	100% (6)
RV lateral wall		
• 3 cardiac cycles		
○ Operator 1	96% (46)	83% (5)
○ Operator 2	96% (46)	100% (6)
• ≥ 1 cardiac cycle		
○ Operator 1	100% (48)	100% (6)
○ Operator 2	100% (48)	100% (6)
Septal wall		
• 3 cardiac cycles		
○ Operator 1	98% (47)	100% (6)
○ Operator 2	98% (47)	100% (6)
• ≥ 1 cardiac cycle		
○ Operator 1	100% (48)	100% (6)
○ Operator 2	100% (48)	100% (6)
RV FAC		
○ Operator 1	100% (48)	83% (5)
○ Operator 2	100% (48)	100% (6)

STE = speckle tracking echocardiography; RV FAC = right ventricular fractional area change

Table S3: Inter-observer reproducibility

	ARDS patients				Control patients		
	ICC coefficient [95% CI]	No of pairs	P value		ICC coefficient [95% CI]	No of pairs	P value
RV Inferior LSS	0.81 [0.68;0.89]	47	<0.001		0.56 [-0.20;0.92]	6	0.08
RV Lateral LSS	0.76 [0.58;0.86]	48	<0.001		0.54 [-0.14;0.91]	6	0.05
RV Septal LSS	0.81 [0.68;0.89]	48	<0.001		0.74 [-0.04;0.96]	6	0.04
RV Global LSS	0.87 [0.72;0.93]	47	<0.001		0.81 [-0.02;0.98]	6	0.002
Global systolic strain rate	0.91 [0.84;0.95]	47	<0.001		0.94 [0.67;0.99]	6	0.001
RV FAC	0.57 [0.35;0.73]	48	0.001		0.19 [-0.94;0.88]	6	0.37
TAPSE	0.88 [0.78;0.93]	48	<0.001				
S’	0.94 [0.89;0.97]	48	<0.001				

RV = right ventricle; LSS = longitudinal systolic strain; FAC = fractional area change; TAPSE = Tricuspid annular plane systolic excursion; S’ = Peak systolic velocity of tricuspid annulus by pulsed wave Doppler tissue imaging

Table S4: Diagnostic value of STE-derived parameters in discriminating RV dysfunction diagnosed by conventional parameters using classic cut-off values (ref 7)

	TAPSE (cut-off 17mm)			S' (cut-off 9.5cm/s)			RV FAC (cut-off 35%)			ED RV:LV >0.6 and at least 1 abnormal conventional parameter		
STE-derived parameters of RV function	AUROC	95% CI	p	AUROC	95% CI	p	AUROC	95% CI	p	AUROC	95% CI	p
RV inferior LSS	0.726	0.574 – 0.878	**0.009**	0.729	0.544 - 0.915	**0.03**	0.776	0.609 - 0.944	**0.003**	0.697	0.547 - 0.847	**0.02**
RV Lateral LSS	0.722	0.573 - 0.872	**0.01**	0.801	0.656 - 0.945	**0.005**	0.811	0.648 - 0.974	**0.001**	0.724	0.579 - 0.869	**0.008**
RV Septal LSS	0.685	0.519 - 0.852	**0.03**	*0.658*	*0.416 - 0.900*	*0.14*	*0.604*	*0.400 - 0.809*	*0.262*	*0.625*	*0.462 - 0.788*	*0.14*
RV Global LSS	0.759	0.614 – 0.904	**0.002**	0.772	0.593 - 0.952	**0.01**	0.777	0.605 - 0.949	**0.003**	0.724	0.577 - 0.871	**0.008**
RV Global LSR	*0.638*	*0.462 - 0.813*	*0.11*	*0.758*	0.554 - 0.962	**0.02**	0.769	0.618 - 0.920	**0.004**	0.669	0.516 - 0.823	**0.04**

AUROC = area under Receiver Operating Characteristic; RV = right ventricle; STE = speckle tracking echocardiography; LSS = longitudinal systolic strain; LSR = longitudinal systolic strain rate; TAPSE = Tricuspid annular plane systolic excursion; FAC = Fractional area change; S' = Peak systolic velocity of tricuspid annulus by pulsed wave Doppler tissue imaging; ED RV:LV = end diastolic right ventricular over left ventricular diameter ratio.

Table S5: Correlation between STE-derived and conventional parameters of RV function in ARDS patients

		TAPSE		S’		RV FAC	
		Correlation	p	Correlation	p	Correlation	p
STE-derived parameters of RV function							
	RV inferior LSS	r = -0.337	0.019	r = -0.294	0.042	r = -0.362	0.012
	RV Lateral LSS	r = -0.424	0.003	r = -0.464	0.001	r = -0.393	0.006
	RV Septal LSS	r = -0.321	0.026	r = -0.203	0.166	r = -0.168	0.254
	RV Global LSS	r = -0.437	0.002	r = -0.401	0.005	r = -0.393	0.006
	Global systolic strain rate	ρ = -0.314	0.030	ρ = -0.630	<0.001	ρ = -0.469	0.001
Conventional parameters							
	TAPSE						
	S’	r = 0.525	<0.001				
	RV FAC	r = 0.394	0.006	r = 0.217	0.138		

RV = right ventricle; STE = speckle tracking echocardiography; LSS = longitudinal systolic strain; TAPSE = Tricuspid annular plane systolic excursion; FAC = Fractional area change; S’ = Peak systolic velocity of tricuspid annulus by pulsed wave Doppler tissue imaging

Table S6: Cut-off values for STE-derived parameters derived from control patients' echocardiography characteristics

	Control patients	Cut-offs (mean ± 2SD)
RV STE-derived parameters		
• RV Inferior LSS %	-25.70 ± 4.07	-17.6
• RV Lateral LSS %	-27.66 ± 4.94	-17.8
• RV Septal LSS %	-19.47 ± 4.19	-11.1
• RV Global LSS %	-24.27 ± 3.44	-17.4
• Global systolic strain rate s^{-1}	-1.76 ± 0.37	-1.02
